# Could Sensory Differences Be a Sex-Indifferent Biomarker of Autism? Early Investigation Comparing Tactile Sensitivity Between Autistic Males and Females

**DOI:** 10.1007/s10803-022-05787-6

**Published:** 2022-10-22

**Authors:** Magdalini Asaridou, Ericka L. Wodka, Richard A. E. Edden, Stewart H. Mostofsky, Nicolaas A. J. Puts, Jason L. He

**Affiliations:** 1 Social, Genetic and Developmental Psychiatric Centre, Institute of Psychiatry, Psychology and Neuroscience, King’s College London, London, UK; 2 Center for Autism and Related Disorders, Kennedy Krieger Institute, Baltimore, MD USA; 3 Department of Psychiatry and Behavioral Sciences, The Johns Hopkins University School of Medicine, Baltimore, MD USA; 4 Russell H. Morgan Department of Radiology and Radiological Science, The Johns Hopkins University School of Medicine, Baltimore, MD USA; 5 F. M. Kirby Research Center for Functional Brain Imaging, Kennedy Krieger Institute, Baltimore, MD USA; 6 Center for Neurodevelopmental and Imaging Research, Kennedy Krieger Institute, Baltimore, MD USA; 7 Department of Neurology, The Johns Hopkins University School of Medicine, Baltimore, MD USA; 8 Department of Forensic and Neurodevelopmental Sciences, Sackler Institute for Translational Neurodevelopment, Institute of Psychiatry, Psychology, and Neuroscience, King’s College London, London, UK; 9 MRC Centre for Neurodevelopmental Disorders, King’s College London, London, UK

**Keywords:** Autism, Sensory, Tactile, Sex-differences, Psychophysics

## Abstract

Sensory differences are highly prevalent in autistic individuals. However, few studies have compared their presentation between autistic males and autistic females. We used psychophysics to assess and compare tactile perceptual sensitivity between autistic and non-autistic boys and girls aged between 8 and 12 years of age. While there were sex-differences of amplitude discrimination, frequency discrimination and order judgement thresholds, these sex-differences were not autism-specific. Mean RTs and detection thresholds were elevated in autism but were comparable between the sexes. Tactile sensitivity measures that are elevated in autism but are otherwise comparable between autistic males and autistic females suggest the possibility that certain sensory features could be used as sex-indifferent markers of autism. Further investigation with larger and more representative samples should be conducted before any stronger conclusions are made.

**Supplementary Information:**

The online version contains supplementary material available at 10.1007/s10803-022-05787-6.

## Introduction

Autistic individuals^1^ tend to be males rather than females,^2^ with there being approximately one female for every four males diagnosed with an autism spectrum condition (Baio et al., 2018; Baird et al., 2006; Fombonne, 2009; Loomes et al., 2017; Mandy et al., 2012). Hypotheses explaining the sex imbalance of autism are generally split into those that argue for biological explanations and those that argue for non-biological, sociological explanations (Greenberg et al., 2018). With respect to the latter, many have suggested that autistic females are currently being underdiagnosed due to the diagnostic criteria having a “male bias” (for some relevant reviews, see Baron-Cohen, 2002; Ferri et al., 2018; Kirkovski et al., 2013; Lai et al., 2015). Over and above the possible bias in the diagnostic criteria, the “core” symptoms of autism (i.e., difficulties with social communication and the presence of restricted and repetitive behaviours) appear to be both qualitatively (Hiller et al., 2014) and quantitatively (Mandy et al., 2012) different between autistic males and autistic females. Importantly, while the core features of autism have been scrutinised over their differences in presentation between autistic males and autistic females, the sensory features of autism, which fall under the domain of restricted and repetitive patterns of behaviours in both the Diagnostic and Statistical Manual of Mental Disorders 5th edition [DSM-5; (American Psychiatric Association, 2013)] and the International Classification of Diseases 11th edition [ICD-11; (World Health Organization, 2018)], have received far less interest regarding whether they present similarly or differently between the sexes.

Studies having compared sensory features between autistic males and autistic females have typically done so under the umbrella of restricted and repetitive behaviours in which a broader range of behaviours unrelated to sensory processing are encapsulated. Since studies typically report total rather than subdomain scores, investigation of sex-differences under restricted and repetitive behaviours tells us very little about whether sensory features present similarly (or differently) between the sexes. There are also very few studies which have directly compared sensory features between autistic males and autistic females using purpose-built measures. Those that exist have typically compared ‘sensory reactivity’ (Aykan et al., 2020; Bitsika et al., 2020; Kumazaki et al., 2015; Lai et al., 2011; Osório et al., 2021), which describes the presence or absence of affective/behavioural reactions to sensory input. The results of studies comparing sensory reactivity between autistic males and autistic females are mixed. Most studies suggest greater sensory reactivity in autistic females than autistic males (Bitsika et al., 2020; Kumazaki et al., 2015; Lai et al., 2011; Osório et al., 2021). However, in contrast, a meta-analysis containing 4606 autistic individuals found that sex was not a significant moderator of sensory features in autism (Ben-Sasson et al., 2019).

In comparison to studies of sex-differences of sensory reactivity, only one study has investigated potential sex-differences of perceptual sensitivity (Tavassoli et al., 2014). Perceptual sensitivity refers to how well one can detect, discriminate, and judge the low-level characteristics of sensory stimuli. Differences of perceptual sensitivity between autistics and non-autistics are well recognised and reported on (see Robertson & Baron-Cohen, 2017), particularly in the tactile domain (Blakemore et al., 2006; C. Cascio et al., 2008; C. J. Cascio et al., 2012; Espenhahn et al., 2022; Foss-Feig et al., 2012; He et al., 2021a, 2021b; O’Riordan & Passetti, 2006; Puts et al., 2014; Sapey-Triomphe et al., 2019; Tavassoli et al., 2016; Tommerdahl et al., 2007a, 2007b; Tommerdahl et al., 2008). Using the Sensory Perception Quotient (SPQ), which contains questions that probe basic perception (e.g., ‘I would be able to tell when an elevator/lift started moving’), Tavassoli and colleagues reported that autistic females had greater perceptual sensitivity than autistic males, providing some evidence of there being a sex-difference of perceptual sensitivity in autism. While questionnaire measures of perception can be useful, more accurate comparisons of perception require performance-based assessments.

To our knowledge, there have been no studies that have used performance-based assessments to investigate potential sex-differences of perceptual sensitivity in autism. Given this, we were interested in using our existing psychophysical data to compare tactile perceptual sensitivity between autistic males and autistic females. The data contained performance outcomes of autistic and non-autistic children with and without co-occurring attention-deficit/hyperactivity disorder (ADHD) who had completed a psychophysical battery assessing tactile perception. The battery included protocols assessing mean response times (RTs) to, and the detection, discrimination, and order judgement of tactile stimulation. Using these data, we have previously demonstrated that tactile sensitivity differed between autistic and non-autistic children, with autistic children showing higher detection, discrimination and order judgement thresholds than typically developing controls (He et al., 2021a, 2021b; He et al., 2021a, 2021b). In the current study, we again compared autistic and non-autistic children on tactile sensitivity, but additionally investigated the moderating effects of sex.

### Methods

The data presented in this study fall under the ethical approval of the Kennedy Krieger Institute and the Johns Hopkins School of Medicine Institutional Review Boards. A caregiver of each child who participated in testing provided written informed consent on the child's behalf and children provided assent.

### Participants

The original sample consisted of 322 participants, of which 112 were autistic males, 18 were autistic females, 137 were typically developing males and 55 were typically developing females. To ensure that comparisons were made using demographically comparable groups, we employed the “MatchIt” R package to perform greedy nearest neighbour matching using propensity scores (Stuart et al., 2011). A comprehensive description of this matching process is provided in Supplementary Materials. After matching, the final sample consisted of a smaller but more demographically comparable 36 autistic males, 18 autistic females, 34 typically developing males and 20 typically developing females. Relevant descriptive statistics of each group can be found in Table 1.

**Table 1:** Descriptive statistics by diagnosis and sex

Statistic	ASC							TDC						p	p_*between*_
	Males			Females			p	Males			Females				
	N	Mean	St. dev	N	Mean	St. dev		N	Mean	St. dev	N	Mean	St. dev		
Age	36	10.76	1.32	18	10.8	1.39	0.917	34	10.47	1.08	20	10.34	1.14	0.689	0.135
WISC-IV FSIQ	23	101.91	13.25	12	105.08	14.94	0.543	23	105.3	11.3	15	108.53	10.41	0.373	0.225
WISC-V FSIQ	13	101.31	19.83	6	91.33	18.35	0.307	16	103.12	10.07	7	104.43	14.34	0.832	0.296
ADOS—communication	36	2.81	1.41	18	2.67	1.14	0.699	–	–	–	–	–	–	–	–
ADOS—social interaction	36	7.81	2.75	18	7.89	3.31	0.927	–	–	-	–	–	–	–	–
ADOS—RRSB	36	2	1.22	18	2.22	1.4	0.57	–	–	–	–	–	–	–	–
ADOS—Total	36	12.61	3.65	18	12.78	4.04	0.884	–	–	–	–	–	–	–	–
ADI—reciprocal social interaction	23	20.74	6.07	13	18.54	5.74	0.289	–	–	–	–	–	–	–	–
ADI—verbal communication	24	16.54	4.29	13	13.46	3.48	0.025	–	–	–	–	–	–	–	–
ADI—RRSB	24	6.58	2.36	13	5.23	1.74	0.056	–	–	–	–	-	–	-	–
ADI—Total	24	2.92	1.18	13	3.08	1.19	0.698	–	–	–	–	–	–	–	–
Conners 3—total hyper	32	79.84	11.5	15	78.2	13.25	0.683	25	47.96	10.8	16	48.31	7.43	0.902	0
Conners 3—total inattention	32	76.62	11.12	15	79.2	11.6	0.479	25	48.8	9.61	16	49.5	10.02	0.826	0
Conners—total hyper	6	65.67	14.47	4	76	16.49	0.348	12	46.17	4.49	4	45.75	3.5	0.854	0.001
Conners—total restless/impulsive	6	69.5	7.94	4	76	16.67	0.508	12	45.08	4.5	4	44.5	1.73	0.715	0
Conners—inattention	6	71.17	10.17	4	73.75	9.98	0.703	12	44.67	4.66	4	43.5	2.38	0.53	0
SPM balance/motion	21	58.71	8.41	11	60.18	6.43	0.587	23	44.26	5.14	11	46.27	6.99	0.408	0
SPM body awareness	21	59.05	8.68	11	60.91	8.5	0.566	23	42.57	5.09	11	43.18	5.67	0.763	0
SPM hearing	21	67.38	5.78	11	64.18	10.34	0.358	23	45.22	5.05	11	44.73	5.73	0.811	0
SPM planning/ideas	21	65.24	6.07	11	61.45	10.87	0.303	23	47.7	5.87	11	45	5.46	0.203	0
SPM social participation	21	65.52	5.57	11	63.73	8.28	0.528	23	45.87	6.57	11	40.55	1.21	0.001	0
SPM total sensory systems	21	64	5.27	11	63.09	8.22	0.744	23	44.61	4.84	11	43.91	5.24	0.713	0

*WISC-IV* Weschler Intelligence Scale – Fourth Edition, *WISC-5* Weschler Intelligence Scale – Fifth Edition, *FSIQ* Full Scale Intelligence Quotient, *ADOS* Autism Diagnostic Observation Schedule, *ADI* Autism Diagnostic Interview (Revised Edition), *RRSB* Restricted Repetitive Stereotyped Behaviours, *Conners 3* Conners 3rd Edition, *SPM* Sensory Processing Measure, *N* Number of Participants, *M* Mean, *SD* Standard Deviation. Note that some children may have completed both versions of a measure (e.g., WISC-IV and WISC-V). p-values provided come from Welch’s t-test (which differs from the standard Pearson’s t-test in that it does not assume equal variance between groups). We caution readers to the take note of the low number of autistic females who completed the SPM (n = 11) and to not overinterpret any group differences (or lack thereof).

### Recruitment, Screening, and General Exclusion Criteria

Recruitment of participants took place in local schools and through advertisements, advocacy organizations, and medical clinics. Screening interviews were conducted via telephone with parents. Exclusion criteria included children with a history of seizures, brain injury or other neurological disorders or illnesses. Participating children had to be able to understand and follow task instructions. For this reason, children with intellectual disabilities were excluded. Children prescribed stimulant medication were asked to stop taking the medication the day before and on the day of the testing.

### Diagnostic Criteria

#### Autism

All autistic children in this study met the 4th and/or 5th edition of the Diagnostic and Statistical Manual of Mental Disorders’ (DSM) criteria for an autism spectrum condition (ASC). Diagnosis was confirmed using the Autism Diagnostic Observation Schedule-Generic [ADOS-G; (Lord et al., 2000)] or Autism Diagnostic Observation Schedule Second Edition [ADOS 2; (Lord et al., 2009)] and the Autism Diagnostic Interview-Revised [ADI-R; (Rutter et al., 2003)]. The ADOS scores used throughout represent ADOS-G scores or the ADOS-2 scores that are comparable to those generated in the ADOS-G.

Intellectual ability was assessed with the WISC-IV and/or WISC-V. The Diagnostic Interview for Children and Adolescents Fourth Edition [DICA-IV; (Reich, 2000)] and/or Kiddie Schedule for Affective Disorders and Schizophrenia [K-SADS; (Kaufman et al., 1997)] was used to determine whether children met criteria for other psychiatric disorders. To rule out learning disabilities, children also completed the word reading subtest from the Wechsler Individual Achievement Test Second Edition [WIAT-II; (Wechsler, 2005)] and/or the Wechsler Individual Achievement Test Third Edition [WIAT-III; (Wechsler, 2009)]. Final diagnosis was (or diagnoses were) confirmed by S. H. M., a child neurologist with extensive experience in the diagnostic assessment of autistic disorders in both research and clinical settings.

Children with full-scale IQ scores below 80 were excluded from participation unless there was a 12-point or greater index discrepancy, in which case either the Verbal Comprehension Index or Perceptual Reasoning Index (or Fluid Reasoning Index and Visual Spatial Index if the child was assessed using the WISC-V) was required to be over 80 and the lower of the two was required to be over 65. Children diagnosed with an identifiable cause of autism (e.g., Fragile X) were excluded from the study. Children who had a learning disability in reading (determined by a significant discrepancy between full-scale IQ and the WIAT-II or WIAT-III Word Reading score or a Word Reading subtest score below 85) were also excluded.

#### Co-Occurring Conditions

Given the high frequency of co-occurring ADHD in ASC (Antshel et al., 2016), children meeting criteria for ADHD were not excluded from the study. Autistic children who met DSM-IV and/or DSM-5 criteria for ADHD were considered ASC + ADHD. To meet criteria for ADHD, children must have had: (1) a T score of 60 or higher on scale L (DSM-IV: inattentive) or M (DSM-IV: hyperactive-impulsive) on the Conners or Conners 3rd edition when available, or a score of 2 or 3 on at least 6 out of 9 items on the inattentive or hyperactivity/impulsivity scales of the ADHD-Rating Scale-IV (DuPaul et al., 1998) and (2) an ADHD diagnosis on the DICA-IV and/or K-SADS. Further information was obtained through the Conners or Conners 3rd edition Parent and Teacher Rating Scales-Revised: Long Form (ADHD-specific broad behaviour rating scales and the ADHD Rating Scale-IV, home, and school versions). The information was reviewed and then diagnosis was verified by S. H. M., a child neurologist with over two decades of experience in diagnosing ADHD in clinical and research settings. In the ASC group, 41 of the 54 children met criteria for ADHD [28 of the 36 ASC males (~ 77%) and 13 of the 18 (~ 72%) ASC females]. Based on the DICA-IV and K-SADS, two children reported having a social anxiety disorder, four for a generalised anxiety disorder and none for a separation anxiety disorder. None of the children reported having major depression or an unspecified depressive disorder. Only one autistic child reported having a disruptive mood dysregulation disorder. Two autistic children were reported as having an oppositional defiant disorder (both had co-occurring ADHD). One autistic child reported having an obsessive–compulsive disorder.

#### Medication

Thirty-one of the autistic children were either taking or had a history of taking ADHD stimulant medication. Ten children were either taking or had a history of taking antidepressants and only one child was taking an antipsychotic.

### Psychophysical Assessment of Tactile Perceptual Sensitivity

**Fig. 1:**
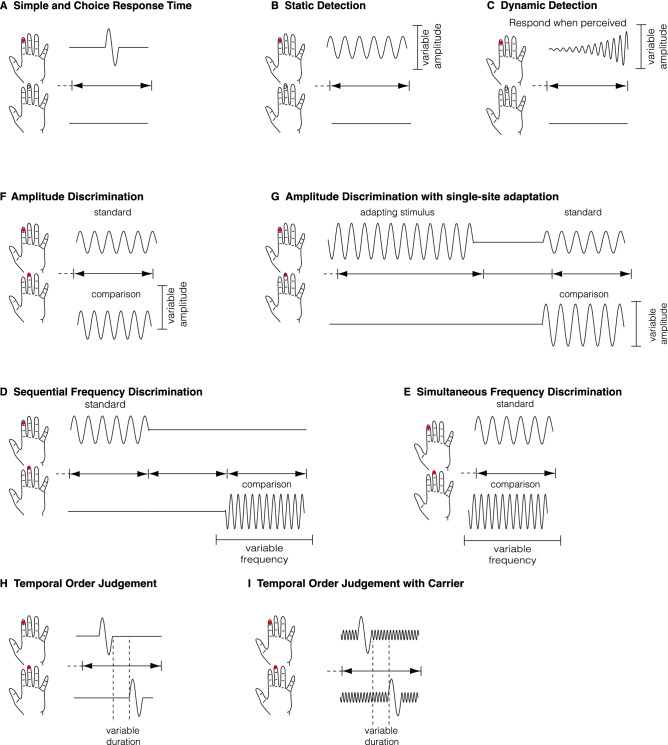
Visual schematic of the vibrotactile protocols. Each participant completed a maximum of ten protocols. Note that only nine protocols are presented, as one of the protocols, namely, Amplitude Discrimination with dual-site adaptation**,** had too few data points for any meaningful comparisons to be made. Protocols were completed sequentially (i.e., the order was not randomised or counter-balanced) and presented in the order of **a** Simple and Choice Response Time (SRT and CRT), **b** Static Detection (SDT), **c** Dynamic Detection (DDT), **d** Amplitude Discrimination (ADT), **e** Amplitude Discrimination with single-site adaptation (ADTssa), **f** Simultaneous Frequency Discrimination (SMFD), **g** Sequential Frequency Discrimination (SQFD), **h** Temporal Order Judgement (TOJ) and **i** Temporal Order judgment with Carrier (TOJwc)**.** Participants who completed the Amplitude Discrimination with dual-site adaptation protocol did so after the ADTssa protocol

Tactile perceptual sensitivity was assessed using two-alternative forced-choice (2AFC) protocols delivered via a Cortical Metrics four-digit tactile stimulator (CM4; Holden et al., 2012). The stimulator consisted of four 5 mm cylindrical probes that delivered vibrotactile stimuli in the form of sinusoidal pulses. All delivered stimuli ranged from 0 to 350 μm and from 0 to 50 Hz. Participants were required to place the fingers of their left hand on each of the four sensors. Only the left digit 2 or the left digit 3 received stimulation. Participants were instructed to respond using the corresponding fingers of their right-hand (i.e., right digit 2 or right digit 3). Task visualisation and parameters of the vibrotactile stimuli were controlled through purpose-made scripts on a portable laptop. To ensure participants understood the protocol prior to beginning, each protocol was preceded by three consecutive practice trials and participants were required to respond accurately to all three trials before beginning the test trials. Participants could request as many breaks as they required between the protocols. See Fig. 1 for a visual schematic of the protocols completed.

The tasks of the battery were designed to be grouped into conceptual pairs. Changes in performance between task pairs are used to inform the functioning of cortical processes reliant on GABA. For example, frequency discrimination was assessed for both sequentially and simultaneously delivered tactile stimuli, and the difference between the conditions was used as an index of lateral inhibition [1]. Below, we describe the protocols within their “condition pairs”. We use acronyms to describe the protocols (e.g., sequential frequency discrimination = SQFD) but refer to thresholds without abbreviations (e.g., sequential frequency discrimination thresholds) for clarity.

### Simple and Choice Response Time

Participants first completed the simple (SRT) and choice (CRT) response time (RT) protocols. These protocols were used to acclimatise participants to the rest of the battery. Both protocols contained 20 trials with an intertrial interval (ITI) of 3 s. In each trial of the SRT and CRT protocols, a suprathreshold stimulus (duration = 40 ms; frequency = 25 Hz; amplitude = 300 µm) was pseudo-randomly delivered to either digit 2 or digit 3 of a participant’s left hand. For the SRT condition, participants were allowed to respond with any finger of their right hand, regardless of which finger had received stimulation on their left hand. In contrast, the CRT condition required participants to specifically respond using the finger on their right hand that corresponded to the finger that had been stimulated on their left hand. For both the SRT and CRT condition, the mean of the median six values were obtained as mean RT. Only correct responses in the CRT were included for estimation of mean RT. The difference in mean RTs between the SRT and CRT conditions were taken as the impact of choice on RTs.

### Static and Dynamic Detection

To assess detection thresholds, participants completed the static detection (SDT) and dynamic detection (DDT) protocols. The SDT condition contained 24 trials with an ITI of 3 s. In each trial of the SDT condition, a suprathreshold stimulus (duration = 500 ms; frequency = 25 Hz; starting amplitude = 20 μm) would be pseudo-randomly delivered to either digit 2 or digit 3 of the participant’s left hand. Participants were required to determine which digit had received the stimulation by responding with the corresponding finger on their right hand. A stepwise adaptive tracking strategy (one-up, one-down for the first ten trials and two-up, one-down for the remaining trials) was used to determine thresholds. The amplitude of the static stimulus increased/decreased in step sizes of 1 μm. Static detection thresholds were determined as the mean amplitude of the final five trials.

The DDT condition contained 7 trials. Each trial began with a variable delay (0–2500 ms). Following the delay, a 25-Hz stimulus of increasing intensity was delivered to either digit 2 or digit 3 of a participant’s left hand. The stimulus intensity would begin at zero and increase at a rate of 2 µm/s. Participants were instructed to respond using the corresponding finger on their right hand “as soon as they felt” the stimulation on their left hand. Dynamic detection thresholds were determined as the mean of the stimulus amplitude at the time of button press across all trials.

We have previously demonstrated that detection thresholds are higher for stimuli of dynamically increasing amplitudes compared to those with static amplitudes (He et al., 2021a, 2021b; Mikkelsen et al., 2020; Puts et al., 2013). Higher detection thresholds for dynamic than static stimuli are thought to reflect feedforward inhibitory functioning. Individual differences in the change in detection thresholds was estimated as percentage change (100 subtracted from one’s detection threshold determined in the DDT condition divided by their detection threshold determined in the SDT condition, multiplied by 100).

### Amplitude Discrimination with and Without Single-Site Adaptation

To determine amplitude discrimination thresholds, participants completed the amplitude discrimination (ADT) and ADT with single-site adaptation (ADTssa) protocols. Both conditions contained 24 trials separated by an ITI of 3 s. In each trial of both protocols, stimuli were delivered to digit 2 and digit 3 of a participant’s left hand simultaneously. One finger received the standard stimulus (duration = 500 ms, frequency = 25 Hz, amplitude = 100 μm) while the other received the comparison stimulus (duration = 500 ms; frequency = 25 Hz; starting amplitude = 200 μm). The fingers receiving the standard/comparison stimuli were pseudo-randomly determined. Participants were asked to choose which of the two simultaneously delivered stimuli had the higher amplitude. In the ADTssa condition, 500 ms prior to the simultaneous stimulation, a single-site adaptation stimulus (duration = 1000 ms; frequency = 25 Hz; amplitude = 100 µm) was delivered to the digit receiving the higher amplitude. A stepwise adaptive tracking strategy (one-up, one-down for the first ten trials and two-up, one-down for the remaining trials) was used to determine thresholds. In both conditions, the amplitude of the comparison stimulus increased/decreased with a step size of 10 μm. Amplitude discrimination thresholds were determined as the mean amplitude difference between the standard and comparison stimulus of the final five trials. Individual differences in the change in amplitude discrimination thresholds were estimated as percentage change (100 subtracted from one’s amplitude discrimination threshold determined in the ADT condition divided by their amplitude discrimination threshold determined in the ADTssa condition, multiplied by 100).

We have previously shown that amplitude discrimination thresholds determined using the ADTssa condition are higher than those determined using the ADT condition (Puts et al., 2013). The adapting stimulus delivered to the finger receiving the higher amplitude is thought to reduce the perceived intensity of the subsequent stimuli by reducing the firing rate of task-relevant neurons through inhibitory mechanisms (Tommerdahl et al., 2007a, 2007b; Zhang et al., 2011). A greater increase in amplitude discrimination thresholds from the ADT to ADTssa condition is therefore thought to reflect less adaptation.

### Sequential and Simultaneous Frequency Discrimination

Frequency discrimination thresholds were assessed using the sequential (SQFD) and simultaneous frequency discrimination (SMFD) protocols. Both conditions contained 20 trials separated by an ITI of 5 s. In the SQFD protocol, stimuli were delivered sequentially to digit 2 and digit 3 of the participant’s left hand [inter-stimulus interval (ISI) = 500 ms]. In the SMFD protocol, the stimuli were delivered simultaneously. In both conditions, one finger received the standard stimulus (duration = 500 ms; frequency = 30 Hz; amplitude = 200 µm) while the other received the comparison stimulus (duration = 500 ms; initial frequency = 40 Hz; amplitude = 200 µm). The fingers receiving the standard/comparison stimuli were pseudo-randomly determined. Participants were asked to choose which finger had received the higher frequency or “faster” stimulus. A stepwise adaptive tracking strategy (one-up, one-down for the first ten trials and two-up, one-down for the remaining trials) was used to determine thresholds. In both conditions, the frequency of the comparison stimulus increased/decreased with a step size of 1 Hz. Frequency discrimination thresholds were determined as the mean amplitude difference between the standard and comparison stimulus of the final five trials. Individual differences in the change in frequency discrimination thresholds were estimated as percentage change (100 subtracted from one’s frequency discrimination threshold determined in the SMFD condition divided by their frequency discrimination threshold determined in the SQFD condition, multiplied by 100).

Discriminating between simultaneously delivered stimuli requires separation of spatially distinct signals before they can be compared, a process likely to depend on lateral inhibition. In contrast, discriminating between sequentially delivered stimuli does not require lateral inhibition, or at least requires less lateral inhibition, because the properties of a stimuli can be encoded separately. We have previously demonstrated that individuals are typically better at discriminating between the frequencies of sequentially rather than simultaneously delivered stimuli (He et al., 2021a, 2021b; Mikkelsen et al., 2020; Puts et al., 2013). Individual differences in the increase in frequency discrimination thresholds from the SQFD to SMFD conditions were used to infer individual differences of lateral inhibitory functioning.

### Temporal Order Judgement with and Without Carrier

To determine tactile order judgement sensitivity, participants completed the temporal order judgement (TOJ) and temporal order judgement with carrier stimulus (TOJwc) protocols. Both protocols contained 20 trials delivered with an ITI of 5 s. In both conditions, two single-cycle vibrotactile pulses (duration = 40 ms; frequency = 25 Hz; amplitude = 200 μm) were delivered to digit 2 and digit 3 of a participant’s left hand (initial ISI = 150 ms). While we recognise that tactile TOJ protocols often involve stimulation to fingers on separate hands (Miyazaki et al., 2016; Tommerdahl et al., 2007a, 2007b), due to the other protocols only requiring stimulation of fingers on one hand, we persisted with a one-handed setup for consistency. The finger receiving the first stimulation was pseudo-randomly determined. Participants were asked to which finger had received the first pulse. In the TOJwc condition, a 25-Hz, 20 µm synchronous carrier stimulus was delivered to both fingers throughout the duration of each trial (1 s). A stepwise adaptive tracking strategy (one-up, one-down for the first ten trials and two-up, one-down for the remaining trials) was used to determine thresholds. In both conditions, the ISI of the comparison stimulus increased/decreased with a step size of 10% of the current ISI. Temporal order judgement thresholds were determined as the mean amplitude difference between the standard and comparison stimulus of the final five trials. Individual differences in the change in temporal order judgement thresholds were estimated as percentage change (100 subtracted from one’s temporal order judgement threshold determined in the TOJwc condition divided by their temporal order judgement threshold determined in the TOJ condition, multiplied by 100).

The low amplitude carrier stimulus delivered throughout the temporal order judgement protocol with carrier protocol is thought to synchronize the neuronal activity between the two fingers, making it more difficult to temporally separate afferent signals. This stimulus driven synchronization has been shown in previous studies and is thought to reflect local connectivity within the somatosensory cortex (Tommerdahl et al., 2008).

### Statistical Analyses

All data were processed using the R programming language in R studio (version 4.0.3). Performance on the tactile protocols were processed using a custom in-house package that is openly available at: https://github.com/HeJasonL/BATD. Prior to analysis, outcome variables went through outlier removal using the median absolute deviation method using a threshold of 2.5. They were then visually assessed for violations of normality. Data visualization was conducted using the ‘ggplot2’ package.

Whether an effect was meaningful was determined through joint consideration of frequentist p-values, effect sizes and Bayes factors. Effect sizes were assessed using partial Eta-squared (η_p_^2^) estimated using the ‘effect size’ package. Bayes factors were assessed using non-informative Jeffreys’s priors and were estimated using the “BayesFactor” package. For those unfamiliar with Bayes factors, “BF_10_” is used to represent the evidence in support of one hypothesis over the evidence in support of another (e.g., evidence in support of an alternative hypothesis over evidence in support of the null hypothesis). A BF_10_ = 10 means that the evidence for the alternative hypothesis (H_1_) is 10 times stronger than the evidence for the null hypothesis (H_0_), whereas a BF_10_ = 0.10 means the evidence is 10 times stronger for H_0_ than H_1_. Bayes factors can also be used to determine the evidence in support of one model over another. In our study, we used BF_10_ to represent the evidence in support of a model (e.g., a model with Sex included as an independent variable) against the evidence in support of the same model without Sex included as an independent variable.

We ran separate linear models for each of our dependent variables, which were: mean RT, detection thresholds, amplitude discrimination thresholds, frequency discrimination thresholds and temporal order judgement thresholds. For each model, condition pair (“Condition”; IV_1_: Condition 1 and Condition 2), diagnostic group (“Diagnosis”; IV_2_: ASC and TDC) and “Sex” (IV_3_: Male and Female) were included as independent variables. All possible two- and three-way interactions were also included.

The study was intended to be exploratory, so we made no directional hypotheses. However, given our earlier findings with these data (He et al., 2021a, 2021b; He et al., 2021a, 2021b), we expected there to be both main effects of “Condition” and “Diagnosis” on tactile sensitivity. We were more interested in whether there was evidence of any sex-differences, and whether there were any sex-differences specific to autism. Evidence of the former would be indicated by a main effect of Sex, while evidence of the latter would be indicated by two-way or three-way interaction effects containing Sex as an independent variable.

Given the relatively small number of autistic females (N = 18), we were concerned that these analyses were possibly underpowered. We attempted to mitigate this issue by using both frequentist and Bayesian statistics, and by providing results of more direct comparisons between autistic males and autistic females using Welch’s two-sample t-tests (which requires less statistical power than the linear models with interaction effects). The results of these more direct comparisons are included as Supplementary Materials.

## Results

### Simple and Choice RTs

**Fig. 2:**
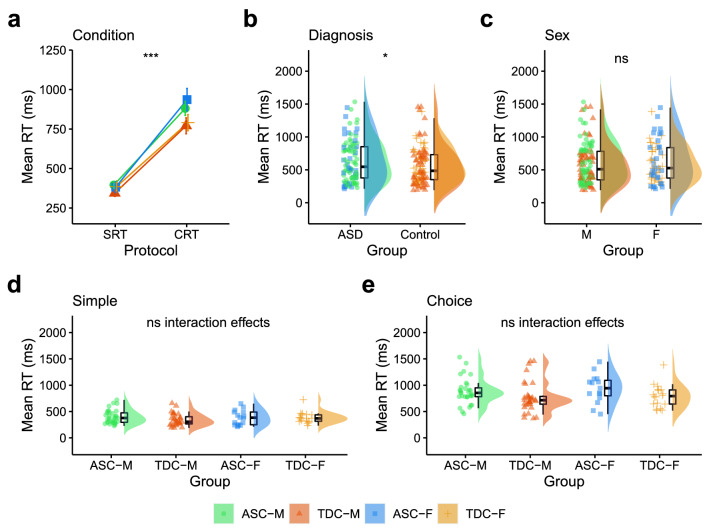
Comparing response times across Condition, Diagnosis and Sex. **a** Mean RTs increased from the Simple to Choice RT condition. **b** Mean RTs in the ASC and TDC groups. **c** Mean RTs in males (M) and females (F). Mean RTs for autistic males (ASC-M), typically developing males (TDC-M), autistic females (ASC-F) and typically developing females (TDC-F) are presented for the **d** Simple and **e** Choice RT conditions separately. *RT* Response Time, *ASC* Autism Spectrum Condition, *TDC* Typically Developing Controls, *F* Female, *M* Male. *p < 0.05, **p < 0.01, *** = t p < 0.001, ns = p > 0.05. Error bars represent standard error

There was a meaningful main effect of Condition [F(1, 172) = 234.76, p < 0.001, η_p_^2^ = 0.11, BF_10_ = 2.51] and Diagnosis [F(1, 172) = 6.12, p = 0.014, η_p_^2^ < 0.01, BF_10_ = 2.61], but not Sex [F(1, 172) = 0.48, p = 0.491, η_p_^2^ = 0.04, BF_10_ = 0.21]. There were no meaningful two-way (all p > 0.166, all BF_10_ between 0.25 and 0.52) or three-way interaction effects (p = 0.526, BF_10_ = 0.015). RTs were longer in the SRT than CRT condition (Fig. 2a). RTs were also longer in the ASC group compared to the TDC group (Fig. 2b). RTs were comparable between the sexes (Fig. 2c). Mean RTs in the SRT (Fig. 2d) and CRT (Fig. 2e) are presented separately for autistic males, typically developing males, autistic females and typically developing females.

### Static and Dynamic Detection Thresholds

**Fig. 3:**
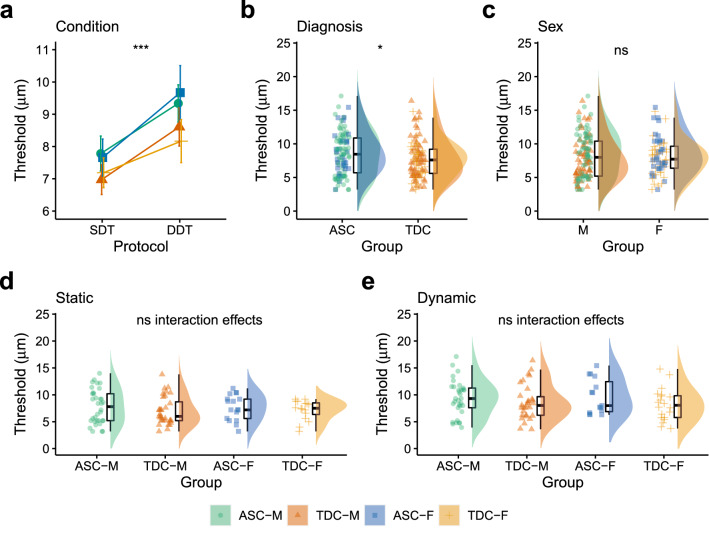
Comparing detection across Condition, Diagnosis and Sex. **a** Detection thresholds increased from the static (SDT) to dynamic detection threshold (DDT) condition. **b** Detection thresholds in ASC and TDC groups. **c** Detection thresholds in males (M) and females (F). Detection thresholds for autistic males (ASC-M), typically developing males (TDC-M), autistic females (ASC-F) and typically developing females (TDC-F) are presented for the **d** SDT and **e** DDT conditions separately. *SDT* Static Detection Threshold, *DDT* Dynamic Detection Threshold, *ASC* Autism Spectrum Condition, *TDC* Typically Developing Controls, *F* Female, *M* Male. *p < 0.05, **p < 0.01, ***p < 0.001, ns = p > 0.05. Error bars represent standard error

There was a meaningful main effect of Condition [F(1, 180) = 16.88, p < 0.001, η_p_^2^ = 0.11, BF_10_ = 322.65] and Diagnosis [F(1, 180) = 4.73, p = 0.031, η_p_^2^ < 0.01, BF_10_ = 1.49], but not Sex [F(1, 180) = 0.01, p = 0.943, η_p_^2^ = 0.04, BF_10_ = 0.16]. There were no meaningful two-way (all p > 0.624, all BF_10_ between 0.22 and 0.31) or three-way interaction effects (p = 0.282, BF_10_ = 0.01). Detection thresholds were higher in the DDT than SDT condition (See Fig. 3a). Detection thresholds were also higher in the ASC than TDC group (Fig. 3b). Detection thresholds were comparable between the sexes (Fig. 3c). Detection thresholds in the SDT (Figs. 3d) and DDT (Fig. 3e) conditions are presented separately for autistic males, typically developing males, autistic females and typically developing females.

### Amplitude Discrimination with and Without Adaptation

**Fig. 4:**
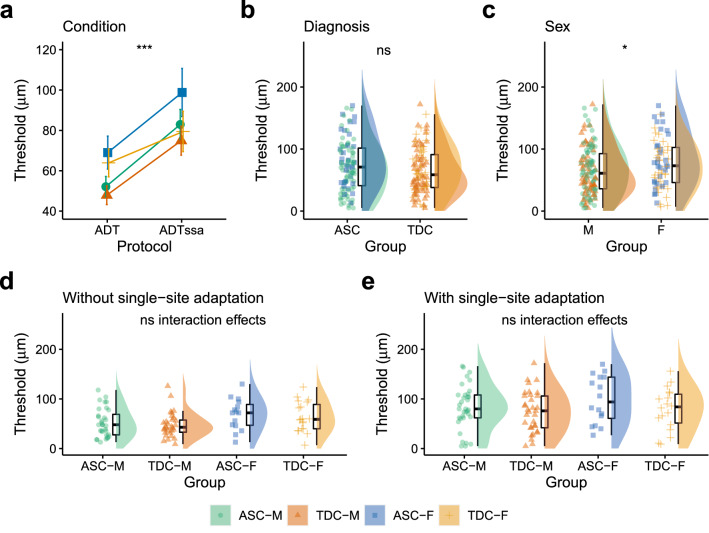
Comparing amplitude discrimination thresholds across Condition, Diagnosis and Sex. **a** Amplitude discrimination thresholds (ADT) increased from the simultaneous ADT condition to the ADT with single-site adaptation stimulus (ADTssa) condition. **b** Amplitude discrimination thresholds in ASC and TDC groups. **c** Amplitude discrimination thresholds in males (M) and females (F). Amplitude discrimination thresholds for autistic males (ASC-M), typically developing males (TDC-M), autistic females (ASC-F) and typically developing females (TDC-F) are presented for the **d** ADT and **e** ADTssa conditions separately. *ADT* Amplitude Discrimination Threshold, *ADTssa* Amplitude Discrimination Threshold with single-site adaptation, *ASC* Autism Spectrum Condition, *TDC* Typically Developing Controls, *F* Female, *M* Male. *p < 0.05, **p < 0.01, ***p < 0.001, ns = p > 0.05. Error bars represent standard error

There was a meaningful main effect of Condition [F(1, 180) = 22.03, p < 0.001, η_p_^2^ = 0.11, BF_10_ = 2769.93] and Sex [F(1, 180) = 5.61, p = 0.019, η_p_^2^ = 0.04, BF_10_ = 2.48] but not Diagnosis [F(1, 180) = 1.22, p = 0.27, η_p_^2^ < 0.01, BF_10_ = 0.26]. There were no meaningful two-way (all p > 0.583, all BF10 between 0.24 and 0.27) or three-way interaction effects (p = 0.553, BF_10_ = 0.01). Amplitude discrimination thresholds were higher in the ADTssa condition than the ADT condition (Fig. 4a). Amplitude discrimination thresholds were comparable between diagnostic groups (see Fig. 4b). Amplitude discrimination thresholds were higher in females than males (Fig. 4c). Amplitude discrimination thresholds in the ADT (Figs. 4d) and ADTssa (Fig. 4e) are presented separately for autistic males, typically developing males, autistic females and typically developing females.

### Sequential and Simultaneous Frequency Discrimination

**Fig. 5:**
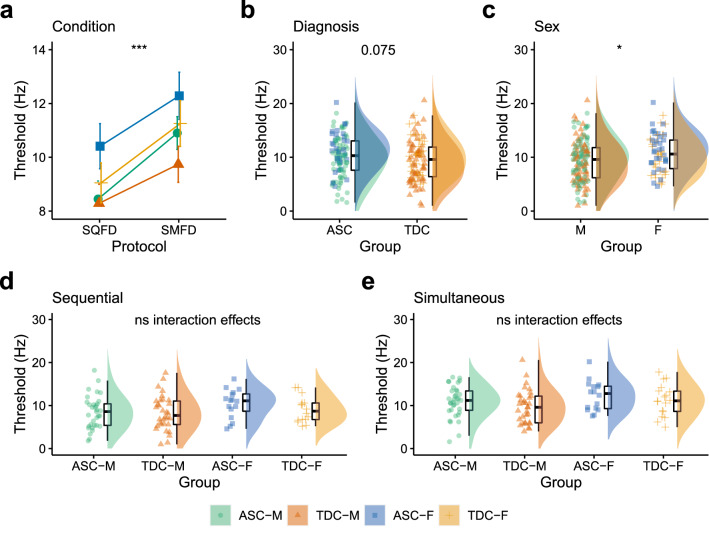
Comparing frequency discrimination thresholds across Condition, Diagnosis and Sex. **a** Frequency discrimination thresholds increased from the sequential frequency discrimination (SQFD) to simultaneous frequency discrimination (SMFD) condition. **b** Frequency discrimination thresholds in the ASC and TDC groups. **c** Frequency discrimination thresholds in males (M) and females (F). Individual data of frequency discrimination thresholds for autistic males (ASC-M), typically developing males (TDC-M), autistic females (ASC-F) and typically developing females (TDC-F) are presented for the **d** SQFD and **e** SMFD conditions separately. *SQFD* Sequential Frequency Discrimination Threshold, *SMFD* Simultaneous Frequency Discrimination Threshold, *ASC* Autism Spectrum Condition, *TDC* Typically Developing Controls, *F* Female, *M* Male. *p < 0.05, **p < 0.01, ***p < 0.001, ns = p > 0.05. Error bars represent standard error

There was a meaningful main effect of Condition [F(1, 184) = 15.55, p < 0.001, η_p_^2^ = 0.11, BF_10_ = 150.21] and Sex [F(1, 184) = 7.04, p = 0.009, η_p_^2^ = 0.04, BF_10_ = 4.55]. The main effect of Diagnosis approached statistical significance, but the BF_10_ suggested more evidence towards the null model than the model suggesting a group difference [Diagnosis: F(1, 184) = 3.22, p = 0.075, η_p_^2^ < 0.01, BF_10_ = 0.67]. There were no meaningful two- (all p > 0.494, all BF_10_ > 0.22 but < 0.27) or three-way (p = 0.359, BF_10_ = 0.01) interaction effects. Frequency discrimination thresholds were higher in the SFD than SQFD condition (Fig. 5a), and higher in the ASC group compared to the TDC group (Fig. 5b). Frequency discrimination thresholds in the SQFD (Figs. 5d) and SMFD (Fig. 5e) conditions are presented separately for autistic males, typically developing males, autistic females and typically developing females.

### Temporal Order Judgement with and Without Carrier

**Fig. 6:**
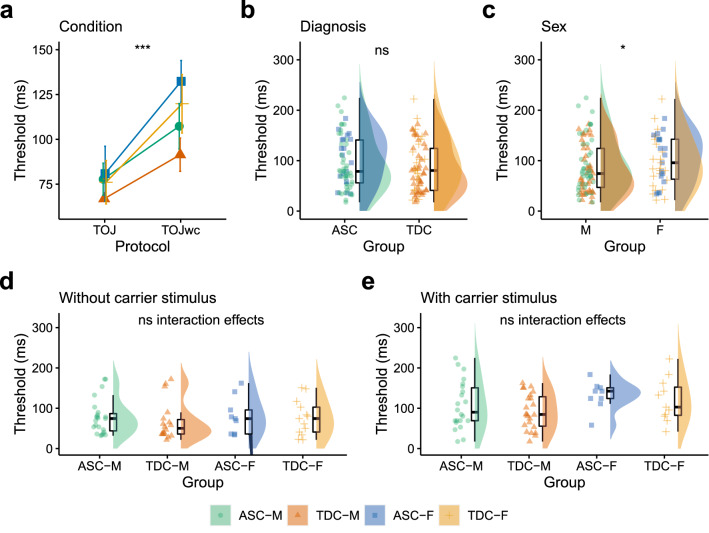
Comparing temporal order judgement discrimination thresholds across Condition, Diagnosis and Sex. **a** Order judgement thresholds increased from the temporal order judgement (TOJ) to TOJ with carrier stimulus (TOJwc) condition. **b** Order judgement thresholds in ASC and TDC groups **c** Order judgement thresholds in males (M) and females (F). Individual data of order judgement thresholds for autistic males (ASC-M), typically developing males (TDC-M), autistic females (ASC-F) and typically developing females (TDC-F) are presented for the **d** TOJ and **e** TOJwc conditions separately. *TOJ* Temporal Order Judgement, *TOJwc* Temporal Order Judgement with carrier, *ASC* Autism Spectrum Condition, *TDC* Typically Developing Controls, *F* Female, *M* Male. *p < 0.05, **p < 0.01, ***p < 0.001, ns = p > 0.05. Error bars represent standard error

There was a meaningful main effect of Condition [F(1, 102) = 13.94, p < 0.001, η_p_^2^ = 0.11, BF_10_ = 78.16] and Sex [F(1, 102) = 5.07, p = 0.026, η_p_^2^ = 0.04, BF_10_ = 2.06] but not Diagnosis [F(1, 102) = 0.05, p = 0.83, η_p_^2^ < 0.01, BF_10_ = 0.20]. There were no meaningful two- (all p > 0.191, BF_10_ > 0.26 but < 0.52) or three-way interaction effects (p = 0.843, BF_10_ = 0.02). Order judgement thresholds were higher in the TOJwc than TOJ condition (Fig. 6a) but were comparable between the diagnostic groups (Fig. 6b). Order judgement thresholds were higher in females compared to males (Fig. 6c). Order judgement thresholds in the TOJ (Figs. 6d) and TOJwc (Fig. 6e) conditions are presented separately for autistic males, typically developing males, autistic females and typically developing females.

## Discussion

There has been persistent interest in investigating whether the core symptoms of autism present similarly between autistic males and autistic females (Ferri et al., 2018; Greenberg et al., 2018; Kumazaki et al., 2015; Mandy et al., 2012; Werling & Geschwind, 2013). While there have been studies comparing difficulties with social communication and restricted/repetitive behaviours between autistic males and autistic females (see Ferri et al., 2018 for a more recent review), very few studies have specifically investigated whether the sensory differences of autism present similarly or differently between the sexes (Bitsika et al., 2018; Kumazaki et al., 2015; Lai et al., 2011; Osório et al., 2021; Tavassoli et al., 2014). Here, we used our existing data to retrospectively explore whether tactile sensitivity was comparable between autistic males and autistic females.

### Higher Discrimination and Order Judgement Thresholds in Females than Males

Across our analyses, there were no meaningful two or three-way interaction effects. While we expected the identified main effects of Condition and Group, we had no expectations with regard to the main effect of Sex or any of the interaction effects which included Sex. Given this, we were surprised to have identified sex-differences of amplitude discrimination, frequency discrimination and order judgement thresholds. Indeed, regardless of diagnosis, amplitude discrimination, frequency discrimination and order judgement thresholds were higher in females compared to males, suggesting less tactile sensitivity in females than males. We initially suspected that these differences to be due to the males and females having not been matched appropriately. This was because our matching process only matched autistic males against autistic females, typically developing males and against typically developing females, and then the autism group against the control group. However, upon comparing the males and females (collapsing across diagnostic group), we found that the groups had very comparable age (p = 0.815) and FSIQ scores (p = 0.874). The sexes also had comparable scores on all domain and total scores on the Conners (all p > 0.465) and Conners 3rd edition (all p > 0.303). Thus, our finding of lower discrimination and order judgement sensitivity in females compared to males were unlikely due to differences in sample demographics.

While sex differences in tactile perception have been previously reported on (Cohen & Levy, 1986; Geffen et al., 2000; Peters et al., 2009), the methodology and participant demographics have varied substantially between studies. For example, while some studies have reported lower discrimination thresholds (i.e., higher tactile sensitivity) in females compared to males (Chen et al., 1995; Komiyama & De Laat, 2005; Komiyama et al., 2007), others have not (Jacklin et al., 1981; Maaser & Farley, 1989). Discrepancies between studies are possibly due to the different body sites having been tested (e.g., while some studies test fingertips like we did, others have tested heels, lips, and other parts of the body). Given that a comprehensive review of existing studies has not been conducted, drawing firm conclusions about sex differences of touch perception is difficult. Interestingly, while the status sex-differences of low-level tactile perception remains unclear, results of a recent meta-analysis suggests that men and women differ on how they perceive affective touch (Russo et al., 2020). Russo and colleagues found evidence to suggest that women perceive affective touch as more pleasant than men. Hormonal and evolutionary differences related to caregiving and nurturing were put forth as possible explanations for this difference. Future studies exploring how sex-differences of low-level tactile perception is related to sex-differences in the perception of affective touch may be of interest.

#### No Autism-Specific Sex-Differences of Tactile Perceptual Sensitivity

For mean RTs and detection thresholds, there were main effects of Condition in the absence of any main effects of Sex and any Condition by Sex interaction effects. This suggests that although mean RTs and detection thresholds were different between the task condition pairs, the change in mean RTs and detection thresholds across task conditions were similar between the sexes, including between autistic males and autistic females. Similarly, given that there was a main effect of Diagnosis in the absence of any main effect of Sex and any Diagnosis by Sex interaction effects, we can infer that although autistic children had higher mean RTs and detection thresholds compared to controls, mean RTs and detection thresholds were comparable between the sexes, including between autistic males and autistic females.

For amplitude discrimination thresholds, there was a significant main effect of Condition and Sex but not Diagnosis. We believe the lack of a main effect of Diagnosis here was due to statistical power, as we have previously demonstrated higher amplitude discrimination thresholds in autism when using larger samples (He et al., 2021a, 2021b; He et al., 2021a, 2021b; Puts et al., 2014). The presence of a main effect of Condition and Sex in the absence of a Condition by Sex interaction suggest that changes in discrimination thresholds between the task condition pairs were comparable between the sexes. The presence of a main effect of Sex in the absence of a Diagnosis by Sex interaction effect suggest that amplitude discrimination thresholds were equally elevated in females, regardless of diagnostic group (see Fig. 4d and e for reference). Thus, while discrimination thresholds are higher in autistic females compared to autistic males (which we also found evidence for in our more direct comparisons using Welch’s two sample t-tests, see Supplementary Table 3), these sex-differences were not specific to autism.

For frequency discrimination thresholds, there was a significant main effect of Condition, a main effect of Diagnosis which approached statistical significance (p = 0.075) and a main effect of Sex. As with amplitude discrimination thresholds, we have previously shown significantly higher frequency discrimination thresholds in autism with larger samples (He et al., 2021a, 2021b; He et al., 2021a, 2021b; Puts et al., 2014), suggesting that the current analyses were just slightly underpowered to reliably detect the difference between diagnostic groups. The absence of a meaningful two-way interaction between Condition and Sex, and three-way interaction between Condition, Diagnosis and Sex, suggest that changes in frequency discrimination thresholds from the SQFD to SMFD task conditions were equal between males and females, including between autistic males and autistic females. Similarly, the absence of any two-way interaction between Diagnosis and Sex, and the three-way interaction between Condition, Diagnosis and Sex suggest that although frequency discrimination thresholds may be elevated in autism, there was no difference in frequency discrimination thresholds between autistic males and autistic females (this was also supported by our more direct comparisons shown in Supplementary Materials). A similar pattern of results was identified for order judgement thresholds.

#### The Finding of Comparable Tactile Perceptual Sensitivity Between Autistic Males and Autistic Females in the Context of Earlier Work

While differences of tactile sensitivity between individuals on the autism spectrum and their neurotypical counterparts have been identified, the sample demographics, methods used, and results, are mixed. We discuss this at length in Mikkelsen et al., (2018). For example, O’Riordan and Passetti (2006) used pieces of sandpaper to assess children’s ability to discriminate between the roughness of sandpaper and found that although the same autistic children had displayed superior auditory discrimination, they had comparable tactile discrimination sensitivity to their neurotypical counterparts. Blakemore et al., (2006) delivered vibrations at both 30 Hz and 200 Hz to the fingertips of autistic and non-autistic adults to assess vibrotactile discrimination and found that detection thresholds were lower in autistic adults, but only for stimuli delivered at 200 Hz. While the autistic adults did have higher detection thresholds than the non-autistic controls at 30 Hz, the group difference did not reach the conventional cut-off for statistical significance (p = 0.11), this was also in a fairly small sample. Cascio et al., (2008) conducted a multidimensional assessment of tactile perception and found that autistic adults had lower detection thresholds for vibrotactile stimuli (33 Hz) delivered to their forearms. Otherwise, autistic adults had comparable vibrotactile detection thresholds at the thenar palm, comparable detection thresholds for light touch (i.e., contact detection thresholds using von Frey elements) at both the forearm and the thenar palm, and comparable detection thresholds for innocuous warm and cool sensations. Over half a decade later, using a vibrotactile battery of stimuli delivered in the flutter range (25–50 Hz), Puts et al., (2014) found that autistic children had higher detection, amplitude discrimination and order judgement thresholds than controls. The results were broadly replicated and expanded in subsequent papers by Tavassoli et al., (2016), He et al., (2021a, 2021b) and Espenhahn et al., (2022). As it stands, there is strong evidence of altered tactile sensitivity in autism. However, there is also clear discrepancy between the studies which could be due to differences in methodology and sample demographics. While a scoping review of tactile processing in autism exists (Mikkelsen et al., 2018), a narrowly focused review of performance based assessments of tactile perception in autism may be warranted.

As canvassed, there has only been one study which has compared sensory perception between autistic males and autistic females (Tavassoli et al., 2014), and this was done so through the use of self-report questionnaires. Our study is the first to compare sensory perception between autistic males and autistic females using psychophysics. Compared to the work of Tavassoli and colleagues, which did not find differences in touch perception between autistic males and autistic females, our study did identify differences in discrimination and order judgement thresholds, though the sex-differences were not specific to autism. The discrepancy in our findings with those of Tavassoli et al., (2014) highlight that self-report and psychophysical assessments of sensory perception are not necessarily congruent and are possibly testing different constructs. The same could be said when comparing our results to the studies which have compared autistic males and autistic females on sensory reactivity (Aykan et al., 2020; Bitsika et al., 2020; Kumazaki et al., 2015; Lai et al., 2011; Osório et al., 2021), which speaks to the affective appraisal of sensory stimuli rather than low-level perception. Future studies comparing sensory differences between autistic males and autistic females should consider including both measures of sensory perception and sensory reactivity.

#### Strengths and Limitations

We have stressed on the outset that the current study is intended to be exploratory and is limited by the small number of autistic females in the current sample. As mentioned in the methods, we supplemented our frequentist analyses with effect sizes and Bayes factors so that results could be considered on a continuum rather than through a binary of significant versus non-significant. We also included more direct comparisons of tactile sensitivity between autistic males and autistic females using Welch’s t-tests in Supplementary Materials. Given the abovementioned limitations and the fact that was study was an exploration of group differences using existing data, the results of this study should be considered preliminary. Drawing any conclusions about whether tactile sensitivity could be used as a sex-indifferent marker of autism would require replication in a larger sample with greater statistical power. Larger samples would not just increase statistical power, but also how representative the sample of autistic participants is of the broader autistic population. Indeed, in the current study, our sample is confined to autistic children. Future studies with wider-age ranges may shed additional light on sex-differences of tactile sensitivity (or sensory differences more broadly) in autism.

The relatively small number of autistic females in the current sample also limited further exploration of the data. For example, while we reported co-occurring ADHD and ADHD symptomatology, we felt the sample was too small to conduct any further analyses that considered co-occurring similarly, we also measured sensory reactivity using the SPM. However, we did not have enough autistic females complete the SPM to warrant analyses including measures from the SPM.

#### Summary and Conclusion

Based on the results of the current study, it appears that tactile sensitivity does differ between autistic males and autistic females. However, these differences are dependent on the perceptual domain assessed. Our results suggest that there were sex-differences for tactile amplitude discrimination, frequency discrimination and order judgement, but there were no sex-differences for mean RTs to tactile stimulation or tactile detection thresholds. Most importantly, the sex-differences we identified were not specific to autism. That is, the sex-differences we identified were present in both autistics and non-autistics (we infer this based on the lack of any two-way or three-way interaction effects including Sex as a variable).

While further replication and investigation in larger samples is required, our results suggest that some of the sensory differences of autism could be used as a sex-indifferent marker. For example, mean RTs and static detection thresholds were elevated in autism (indicated by a main effect of Diagnosis) but were comparable between autistic males and autistic females (indicated by a non-significant main effect of Sex and the absence of any interaction effects including Sex). If these effects can be replicated in a larger sample, we would have what could function as a sex-indifferent marker of autism. Such a marker would be useful, as autistic females are currently underdiagnosed and the other core symptoms of autism are affected by sex. The identification of sensory features that present comparably between the sexes, but differently between autistics and non-autistics could be used to aid the unbiased diagnosis of autism in females. Still, as stressed, the results of the current study should be considered as preliminary and more effort in comparing sensory features between autistic males and autistic females, especially across different levels of analysis and age groups, is required.

### Supplementary Information

Below is the link to the electronic supplementary material.

Supplementary file1 (DOCX 201 kb)

## Footnotes

1 Identity-first language has been suggested to be the preferred language of the majority of people diagnosed with autism^50,51^.

2 Here, the terms male and female (or sex) are used to refer to the physiological and physical aspects of gender rather than gender identity, self-defined gender, legal or social gender^52^.

## References

[CR1] American Psychiatric Association (2013). Diagnostic and statistical manual of mental disorders (DSM-5®).

[CR2] Antshel KM, Zhang-James Y, Wagner KE, Ledesma A, Faraone SV (2016). An update on the comorbidity of ADHD and ASD: A focus on clinical management. Expert Review of Neurotherapeutics.

[CR3] Aykan S, Gürses E, Tokgöz-Yılmaz S, Kalaycıoğlu C (2020). Auditory processing differences correlate with autistic traits in males. Frontiers in Human Neuroscience.

[CR4] Baio J, Wiggins L, Christensen DL, Maenner MJ, Daniels J, Warren Z, Kurzius-Spencer M, Zahorodny W, Robinson Rosenberg C, White T, Durkin MS, Imm P, Nikolaou L, Yeargin-Allsopp M, Lee L-C, Harrington R, Lopez M, Fitzgerald RT, Hewitt A, Dowling NF (2018). Prevalence of autism spectrum disorder among children aged 8 years—autism and developmental disabilities monitoring network, 11 sites, united states, 2014. MMWR Surveillance Summaries.

[CR5] Baird G, Simonoff E, Pickles A, Chandler S, Loucas T, Meldrum D, Charman T (2006). Prevalence of disorders of the autism spectrum in a population cohort of children in South Thames: The special needs and autism project (SNAP). Lancet.

[CR6] Baron-Cohen S (2002). The extreme male brain theory of autism. Trends in Cognitive Sciences.

[CR7] Ben-Sasson A, Gal E, Fluss R, Katz-Zetler N, Cermak SA (2019). Update of a meta-analysis of sensory symptoms in ASD: A new decade of research. Journal of Autism and Developmental Disorders.

[CR8] Bitsika V, Arnold WA, Sharpley CF (2020). The role of sensory features in mediating associations between autism symptoms and anxiety in boys with autism spectrum disorder. Journal of Autism and Developmental Disorders.

[CR9] Bitsika V, Sharpley CF, Mills R (2018). Sex differences in sensory features between boys and girls with autism spectrum disorder. Research in Autism Spectrum Disorders.

[CR10] Blakemore S-J, Tavassoli T, Calò S, Thomas RM, Catmur C, Frith U, Haggard P (2006). Tactile sensitivity in asperger syndrome. Brain and Cognition.

[CR11] Cascio C, McGlone F, Folger S, Tannan V, Baranek G, Pelphrey KA, Essick G (2008). Tactile perception in adults with autism: A multidimensional psychophysical study. Journal of Autism and Developmental Disorders.

[CR12] Cascio CJ, Moana-Filho EJ, Guest S, Nebel MB, Weisner J, Baranek GT, Essick GK (2012). Perceptual and neural response to affective tactile texture stimulation in adults with autism spectrum disorders. Autism Research.

[CR13] Chen C, Essick G, Kelly D, Young M, Nestor J, Masse B (1995). Gender-, side-and site-dependent variations in human perioral spatial resolution. Archives of Oral Biology.

[CR14] Cohen H, Levy JJ (1986). Sex differences in categorization of tactile stimuli. Perceptual and Motor Skills.

[CR15] DuPaul GJ, Power TJ, Anastopoulos AD, Reid R (1998). ADHD Rating Scale—IV: Checklists, norms, and clinical interpretation (pp. viii, 79).

[CR16] Espenhahn S, Godfrey KJ, Kaur S, McMorris C, Murias K, Tommerdahl M, Bray S, Harris AD (2022). Atypical tactile perception in early childhood autism. Journal of Autism and Developmental Disorders.

[CR17] Ferri SL, Abel T, Brodkin ES (2018). Sex differences in autism spectrum disorder: A review. Current Psychiatry Reports.

[CR18] Fombonne E (2009). Epidemiology of pervasive developmental disorders. Pediatric Research.

[CR19] Foss-Feig JH, Heacock JL, Cascio CJ (2012). Tactile responsiveness patterns and their association with core features in autism spectrum disorders. Research in Autism Spectrum Disorders.

[CR20] Geffen G, Rosa V, Luciano M (2000). Sex differences in the perception of tactile simultaneity. Cortex.

[CR21] Greenberg DM, Warrier V, Allison C, Baron-Cohen S (2018). Testing the empathizing-systemizing theory of sex differences and the extreme male brain theory of autism in half a million people. Proceedings of the National Academy of Sciences.

[CR22] He JL, Oeltzschner G, Mikkelsen M, Deronda A, Harris AD, Crocetti D, Wodka EL, Mostofsky SH, Edden RAE, Puts NAJ (2021). Region-specific elevations of glutamate + glutamine correlate with the sensory symptoms of autism spectrum disorders. Translational Psychiatry.

[CR23] He JL, Wodka E, Tommerdahl M, Edden RAE, Mikkelsen M, Mostofsky SH, Puts NAJ (2021). Disorder-specific alterations of tactile sensitivity in neurodevelopmental disorders. Communications Biology.

[CR24] Hiller RM, Young RL, Weber N (2014). Sex Differences in autism spectrum disorder based on DSM-5 criteria: evidence from clinician and teacher reporting. Journal of Abnormal Child Psychology.

[CR25] Holden JK, Nguyen RH, Francisco EM, Zhang Z, Dennis RG, Tommerdahl M (2012). A novel device for the study of somatosensory information processing. Journal of Neuroscience Methods.

[CR26] Jacklin CN, Snow ME, Maccoby EE (1981). Tactile sensitivity and muscle strength in newborn boys and girls. Infant Behavior and Development.

[CR27] Kaufman J, Birmaher B, Brent D, Rao U, Flynn C, Moreci P, Williamson D, Ryan N (1997). Schedule for affective disorders and schizophrenia for school-age children-present and lifetime version (K-SADS-PL): Initial reliability and validity data. Journal of the American Academy of Child & Adolescent Psychiatry.

[CR28] Kirkovski M, Enticott PG, Fitzgerald PB (2013). A review of the role of female gender in autism spectrum disorders. Journal of Autism and Developmental Disorders.

[CR29] Komiyama O, De Laat A (2005). Tactile and pain thresholds in the intra-and extra-oral regions of symptom-free subjects. Pain.

[CR30] Komiyama O, Kawara M, De Laat A (2007). Ethnic differences regarding tactile and pain thresholds in the trigeminal region. The Journal of Pain.

[CR31] Kumazaki H, Muramatsu T, Kosaka H, Fujisawa TX, Iwata K, Tomoda A, Tsuchiya K, Mimura M (2015). Sex differences in cognitive and symptom profiles in children with high functioning autism spectrum disorders. Research in Autism Spectrum Disorders.

[CR32] Lai M-C, Lombardo MV, Auyeung B, Chakrabarti B, Baron-Cohen S (2015). Sex/gender differences and autism: setting the scene for future research. Journal of the American Academy of Child and Adolescent Psychiatry.

[CR33] Lai, M.-C., Lombardo, M. V., Pasco, G., Ruigrok, A. N., Wheelwright, S. J., Sadek, S. A., Chakrabarti, B., MRC Aims Consortium, & Baron-Cohen, S (2011). A behavioral comparison of male and female adults with high functioning autism spectrum conditions. PLoS ONE.

[CR34] Loomes R, Hull L, Mandy WPL (2017). What is the male-to-female ratio in autism spectrum disorder? A systematic review and meta-analysis. Journal of the American Academy of Child & Adolescent Psychiatry.

[CR35] Lord C, Risi S, Lambrecht L, Cook EH, Leventhal BL, DiLavore PC, Pickles A, Rutter M (2000). The autism diagnostic observation schedule—generic: A standard measure of social and communication deficits associated with the spectrum of autism. Journal of Autism and Developmental Disorders.

[CR36] Lord C, Rutter M, DiLavore P, Risi S, Gotham K, Bishop S (2009). Autism diagnostic observation schedule.

[CR37] Maaser BW, Farley FH (1989). Hemispheric differences in arousability and strength of the nervous system research communications in psychology, psychiatry & behavior.

[CR38] Mandy W, Chilvers R, Chowdhury U, Salter G, Seigal A, Skuse D (2012). Sex differences in autism spectrum disorder: Evidence from a large sample of children and adolescents. Journal of Autism and Developmental Disorders.

[CR39] Mikkelsen M, He J, Tommerdahl M, Edden RAE, Mostofsky SH, Puts NAJ (2020). Reproducibility of flutter-range vibrotactile detection and discrimination thresholds. Scientific Reports.

[CR40] Mikkelsen M, Wodka EL, Mostofsky SH, Puts NAJ (2018). Autism spectrum disorder in the scope of tactile processing. Developmental Cognitive Neuroscience.

[CR41] Miyazaki M, Kadota H, Matsuzaki KS, Takeuchi S, Sekiguchi H, Aoyama T, Kochiyama T (2016). Dissociating the neural correlates of tactile temporal order and simultaneity judgements. Scientific Reports.

[CR42] O’Riordan M, Passetti F (2006). Discrimination in autism within different sensory modalities. Journal of Autism and Developmental Disorders.

[CR43] O’Riordan M, Passetti F (2006). Discrimination in autism within different sensory modalities. Journal of Autism and Developmental Disorders.

[CR44] Osório JMA, Rodríguez-Herreros B, Richetin S, Junod V, Romascano D, Pittet V, Chabane N, Jequier Gygax M, Maillard AM (2021). Sex differences in sensory processing in children with autism spectrum disorder. Autism Research.

[CR45] Peters RM, Hackeman E, Goldreich D (2009). Diminutive digits discern delicate details: Fingertip size and the sex difference in tactile spatial acuity. Journal of Neuroscience.

[CR46] Puts NAJ, Edden RAE, Wodka EL, Mostofsky SH, Tommerdahl M (2013). A vibrotactile behavioral battery for investigating somatosensory processing in children and adults. Journal of Neuroscience Methods.

[CR47] Puts NAJ, Wodka EL, Tommerdahl M, Mostofsky SH, Edden RAE (2014). Impaired tactile processing in children with autism spectrum disorder. Journal of Neurophysiology.

[CR48] Reich W (2000). Diagnostic interview for children and adolescents (DICA). Journal of the American Academy of Child & Adolescent Psychiatry.

[CR49] Robertson CE, Baron-Cohen S (2017). Sensory perception in autism. Nature Reviews Neuroscience.

[CR50] Russo V, Ottaviani C, Spitoni GF (2020). Affective touch: A meta-analysis on sex differences. Neuroscience & Biobehavioral Reviews.

[CR51] Rutter M, Le Couteur A, Lord C (2003). Autism diagnostic interview-revised. Los Angeles, CA: Western Psychological Services.

[CR52] Sapey-Triomphe L-A, Lamberton F, Sonié S, Mattout J, Schmitz C (2019). Tactile hypersensitivity and GABA concentration in the sensorimotor cortex of adults with autism. Autism Research.

[CR53] Stuart EA, King G, Imai K, Ho D (2011). MatchIt: Nonparametric preprocessing for parametric causal inference. Journal of Statistical Software.

[CR54] Tavassoli T, Bellesheim K, Tommerdahl M, Holden JM, Kolevzon A, Buxbaum JD (2016). Altered tactile processing in children with autism spectrum disorder. Autism Research.

[CR55] Tavassoli T, Hoekstra RA, Baron-Cohen S (2014). The sensory perception quotient (SPQ): Development and validation of a new sensory questionnaire for adults with and without autism. Molecular Autism.

[CR56] Tommerdahl M, Tannan V, Cascio C, Baranek G, Whitsel B (2007). Vibrotactile adaptation fails to enhance spatial localization in adults with autism. Brain Research.

[CR57] Tommerdahl M, Tannan V, Holden JK, Baranek GT (2008). Absence of stimulus-driven synchronization effects on sensory perception in autism: Evidence for local underconnectivity?. Behavioral and Brain Functions.

[CR58] Tommerdahl M, Tannan V, Zachek M, Holden JK, Favorov OV (2007). Effects of stimulus-driven synchronization on sensory perception. Behavioral and Brain Functions: BBF.

[CR59] Wechsler D (2005). Wechsler Individual achievement test—second edition (WIAT-II).

[CR60] Wechsler D (2009). Wechsler individual achievement test.

[CR61] Werling DM, Geschwind DH (2013). Sex differences in autism spectrum disorders. Current Opinion in Neurology.

[CR62] World Health Organization (2018). ICD-11 for mortality and morbidity statistics 2018.

[CR63] Zhang Z, Francisco EM, Holden JK, Dennis RG, Tommerdahl M (2011). Somatosensory information processing in the aging population. Frontiers in Aging Neuroscience.

