# Supplementary material for: Could Sensory Differences Be a Sex-Indifferent Biomarker of Autism? Early Investigation Comparing Tactile Sensitivity Between Autistic Males and Females
Source: J Autism Dev Disord. 2022 Oct 22;54(1):239–55. doi: 10.1007/s10803-022-05787-6 (PMC10791919; doi:10.1007/s10803-022-05787-6)
Supplement: Supplementary file 1 — Supplementary file1 (DOCX 201 kb) [file 10803_2022_5787_MOESM1_ESM.docx]

**Could Sensory Differences Be a Sex-Indifferent Biomarker of Autism? Early Investigation Comparing Tactile Sensitivity Between Autistic Males and Females**

Magdalini Asaridou^1^, Ericka L. Wodka^2, 3^, Richard A. E. Edden^4, 5^, Stewart H. Mostofsky^3,6,7,^, Nicolaas A. J. Puts^8,9^ & Jason L. He^8^

*^1^Social, Genetic and Developmental Psychiatric Centre, Institute of Psychiatry, Psychology and Neuroscience, King’s College London*

*^2^Center for Autism and Related Disorders, Kennedy Krieger Institute, Baltimore, MD United States*

*^3^ Department of Psychiatry and Behavioral Sciences, The Johns Hopkins University School of Medicine, Baltimore, MD, United States.*

^4^*Russell H. Morgan Department of Radiology and Radiological Science, The Johns Hopkins University School of Medicine, Baltimore, MD, United States*

*^5^F. M. Kirby Research Center for Functional Brain Imaging, Kennedy Krieger Institute, Baltimore, MD, United States*

*^6^Center for Neurodevelopmental and Imaging Research, Kennedy Krieger Institute, Baltimore, MD, United States*

*^7^ Department of Neurology, The Johns Hopkins University School of Medicine, Baltimore, MD, United States.*

*^8^Department of Forensic and Neurodevelopmental Sciences, Sackler Institute for Translational Neurodevelopment, Institute of Psychiatry, Psychology, and Neuroscience, King's College London, London, UK*

*^9^ MRC Centre for Neurodevelopmental Disorders, King’s College London, London, United Kingdom*

**Keywords:** Autism, sensory, tactile, sex-differences, psychophysics

**Corresponding Author:** Jason L. He

Department of Forensic and Neurodevelopmental Sciences,

Sackler Institute for Translational Neurodevelopment,

Institute of Psychiatry, Psychology, and Neuroscience,

King’s College London, UK

[jason.he@kcl.ac.uk](mailto:jason.he@kcl.ac.uk)

**Matching groups using “MatchIt” functions**

We began with a sample of 322 participants, of which 130 were autistic and 192 were controls. We were interested in maximizing the comparability of relevant demographic variables between a) the autistic males and females, b) the control male and females and c) the autistic and control participants. The demographic variables we were interested in matching were age, sex, full-scale intelligence quotient (FSIQ), autism and ADHD symptomatology. We assessed the comparability on these variables prior to matching. See Supplementary Table 1.

**Supplementary Table 1**

|  | ASC | | | | | | |  | TDC | | | | | |  |  |
| --- | --- | --- | --- | --- | --- | --- | --- | --- | --- | --- | --- | --- | --- | --- | --- | --- |
|  | Males | | | Females | | |  |  | Males | | | Females | | |  |  |
| Statistic | N | Mean | St. Dev. | N | Mean | St. Dev. | *p* |  | N | Mean | St. Dev. | N | Mean | St. Dev. | *p* | *pBetween* |
| Age | 112 | 10.4 | 1.32 | 18 | 10.8 | 1.39 | 0.26205 |  | 137 | 10.23 | 1.24 | 55 | 10.06 | 1.04 | 0.34708 | 0.05803 |
| WISC-IV FSIQ | 60 | 100.25 | 15.65 | 12 | 105.08 | 14.94 | 0.32503 |  | 98 | 115.99 | 13.02 | 41 | 115.51 | 10.41 | 0.81989 | 0 |
| WISC-V FSIQ | 52 | 98.1 | 15.69 | 6 | 91.33 | 18.35 | 0.41995 |  | 57 | 114.18 | 13.2 | 21 | 112 | 10.94 | 0.4661 | 0 |
| FSIQ (WISC-IV + WISC-V) | 112 | 99.25 | 15.63 | 18 | 100.5 | 16.97 | 0.77214 |  | 137 | 115.59 | 12.99 | 55 | 113.85 | 10.67 | 0.34109 | 0 |
| ADOS Communication | 111 | 3.75 | 1.46 | 18 | 2.67 | 1.14 | 0.00132 |  | - | - | - | - | - | - | - | - |
| ADOS Social Interaction | 107 | 8.42 | 2.73 | 18 | 7.89 | 3.31 | 0.52507 |  | - | - | - | - | - | - | - | - |
| ADOS RRSB | 107 | 2.96 | 1.7 | 18 | 2.22 | 1.4 | 0.0545 |  | - | - | - | - | - | - | - | - |
| ADOS Total | 107 | 15.17 | 4.4 | 18 | 12.78 | 4.04 | 0.03075 |  | - | - | - | - | - | - | - | - |
| Conners 3 Hyperactive | 83 | 75.53 | 13.03 | 15 | 78.2 | 13.25 | 0.48005 |  | 88 | 47.51 | 9.15 | 45 | 47.24 | 8.69 | 0.8696 | 0 |
| Conners 3 Inattentive | 83 | 74.3 | 12.16 | 15 | 79.2 | 11.6 | 0.15069 |  | 88 | 47.3 | 8.87 | 45 | 47.47 | 7.64 | 0.90812 | 0 |
| Conners Hyperactive | 32 | 63.12 | 13.05 | 4 | 76 | 16.49 | 0.21726 |  | 58 | 46.52 | 5.25 | 13 | 47.62 | 4.39 | 0.4413 | 0 |
| Conners Inattentive | 32 | 64.06 | 11.16 | 4 | 76 | 16.67 | 0.24891 |  | 58 | 45.41 | 4.77 | 13 | 47.08 | 4.96 | 0.28603 | 0 |
| Conners + Conners 3 Hyper. | 112 | 72.2 | 14.27 | 18 | 78.83 | 13.01 | 0.05906 |  | 137 | 47.09 | 8.01 | 55 | 47.2 | 8.03 | 0.93026 | 0 |
| Conners + Conners 3 Inatt. | 112 | 71.64 | 12.66 | 18 | 78.28 | 11.29 | 0.03205 |  | 137 | 46.75 | 7.63 | 55 | 47.09 | 7.08 | 0.76987 | 0 |

Participants had either WISC-IV or WISC-V assessments. FSIQ is a score that collapses across the measures. This was required due to the “matchit” function not allowing variables with missing data. This reason is also why we have Conners + Conners 3 Hyper and Conners + Conners 3 Inat (which are just scores collapsed across the Conners and Conners 3^rd^ edition). WISC = Weschler Intelligence Scale for Children, ADOS = Autism Diagnostic Observation Scale, N = number of participants, ASC = Autism Spectrum Condition, TDC = Typically Developing Controls.

While the autistic male and females were broadly comparable to begin with, it is noticeable that their ADOS total communication scores were significantly different. Their ADOS total and Conners hyperactivity domain total scores were also approached statistical significance. Given that our primary interest was in comparing tactile sensitivity between autistic males and females, we needed to match the groups on these measures. As with all the matching that we describe here, our aim was to match groups as much as possible while maintaining the sample size (and hence, statistical power).

We used the “matchit” function from the “MatchIt” package, opting to use 1:1 nearest neighbour matching on propensity scores. An overview of how this works can be found here (<https://cran.r-project.org/web/packages/MatchIt/vignettes/MatchIt.html>). Prior to matching, one must decide on a “treated” group and a “control” group. The “treated” group is the group you wish to find matches from the “control” group. We used general linear modelling methods to estimate propensity scores. Propensity scores are a one-dimensional summary of the covariates (i.e., the demographic variables listed in Supplementary Table 1) computed as the predicted probability of being in a designated “treated” group. Each participant in the “treated” group is paired with an available control unit with the nearest propensity score. The participants in the “treated” and “matched” groups are then removed from their groups and the process is repeated (i.e., matching occurs without replacement).

The “matchit” function only allows two groups to be matched at a time. Working with this limitation, we matched the autistic males and females first (step 1), the control males and females second (step 2), and then the autism group with the control group (step 3). The formulas used in the “matchit” argument are provided for each step below. In each step, the female group was treated as the “treated” group while the males were treated as the “control” group. This ensured that the number of females were maximized (which was important since there were more males to females in the sample, particularly in the autism group).

**Formulas for matching steps 1, 2 and 3.**

1. Group ~ age + FSIQ + Conners Total Hyperactivity Score + Conners Total Inattention Score + ADOS Total Score + ADOS Communication Score + ADOS Social Interaction Score + ADOS Stereotyped Score
2. Group ~ age + FSIQ + Conners Total Hyperactivity Score + Conners Total Inattention Score
3. Group ~ age + FSIQ

Given that the controls did not complete the ADOS, they could not be matched based on ADOS scores (hence the difference between steps 1 and 2, and also the lack of ADOS measures in step 3). While we wanted to match the autism and ADHD groups on ADHD symptomatology, this was not possible. This was perhaps expected since even in autistic individuals without co-occurring ADHD, ADHD symptomatology is elevated. Given that the controls could not have co-occurring ADHD, it was expected that there would be few controls matches for the autistic participants with high ADHD scores. We opted to not match the groups on ADHD symptomatology.

We used the built in plotting functions of “MatchIt” to produce plots that show propensity scores on the x-axis, the unmatched “treated” units, matched “treated” units, matched “control” units and unmatched “control” units. Good matching can be discerned by the matched units being far from the unmatched units on the x-axis. Units are jittered to prevent visual overlap.

**Step 1 matching:**

**
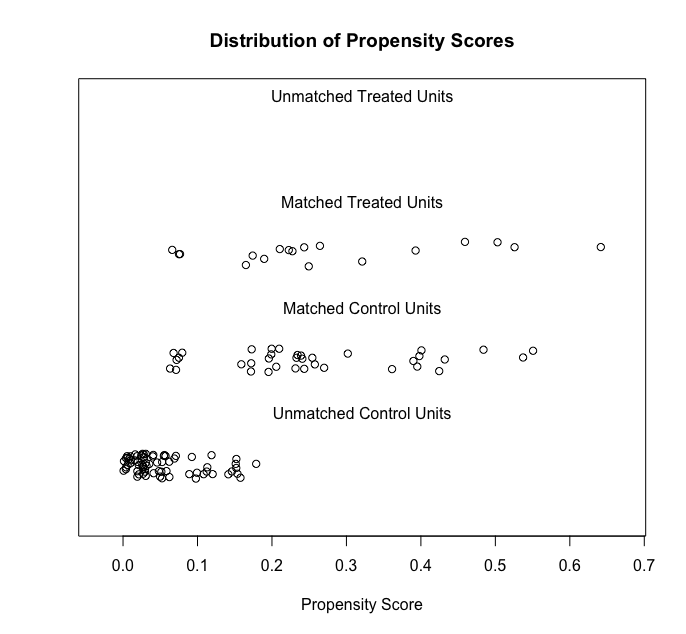
**

**Step 2 matching:**


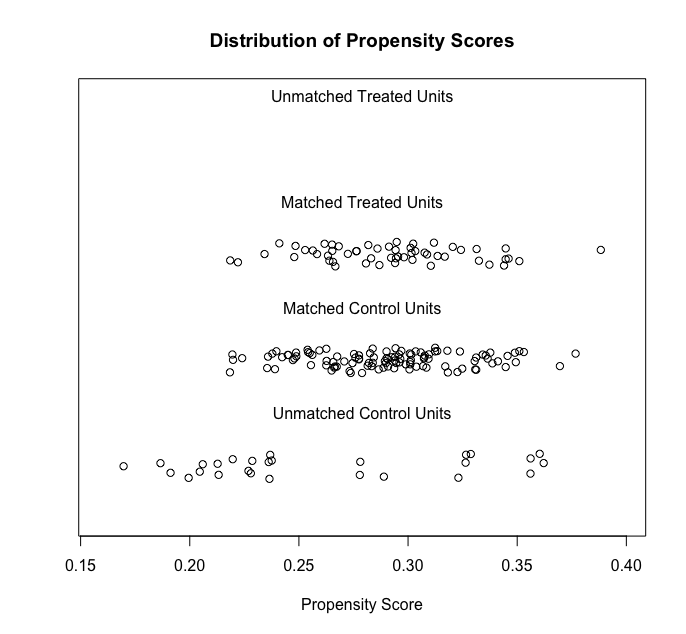


**Step 3 matching:**

**
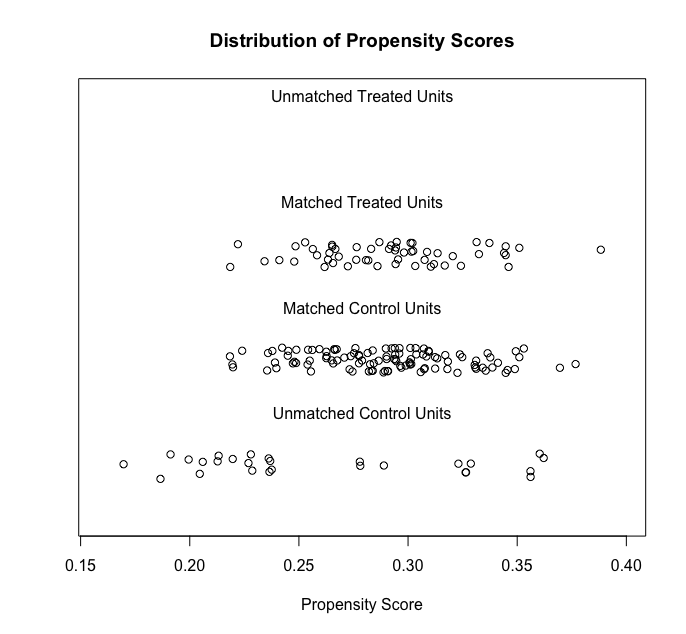
**

The resulting sample, while smaller than the initial sample, is far better matched. As can be discerned in Supplementary Table 2, the autistic males and females are now matched on all of the descriptive statistics (and are far from statistically significantly different). The control males and females were already quite matched so there was little improve. What matters more is the matching between the autism and control groups. As can be discerned, the groups are now much better matched on age and FSIQ. The final sample (N = 108) had 36 autistic males, 18 autistic females, 34 control males and 20 control females. This sample is the sample used for all analyses in the main text.

**Supplementary Table 2**

|  | ASC | | | | | | |  | TDC | | | | | |  |  |
| --- | --- | --- | --- | --- | --- | --- | --- | --- | --- | --- | --- | --- | --- | --- | --- | --- |
|  | Males | | | Females | | |  |  | Males | | | Females | | |  |  |
| Statistic | N | Mean | St. Dev. | N | Mean | St. Dev. | *p* |  | N | Mean | St. Dev. | N | Mean | St. Dev. | *p* | *pBetween* |
| Age | 36 | 10.76 | 1.32 | 18 | 10.8 | 1.39 | 0.91739 |  | 34 | 10.47 | 1.08 | 20 | 10.34 | 1.14 | 0.6893 | 0.13468 |
| WISC-IV FSIQ | 23 | 101.91 | 13.25 | 12 | 105.08 | 14.94 | 0.5428 |  | 23 | 105.3 | 11.3 | 15 | 108.53 | 10.41 | 0.37305 | 0.22451 |
| WISC-V FSIQ | 13 | 101.31 | 19.83 | 6 | 91.33 | 18.35 | 0.30703 |  | 16 | 103.12 | 10.07 | 7 | 104.43 | 14.34 | 0.83234 | 0.29618 |
| FSIQ (WISC-IV + WISC-V) | 36 | 101.69 | 15.66 | 18 | 100.5 | 16.97 | 0.80411 |  | 34 | 104.18 | 10.28 | 20 | 105.9 | 10.59 | 0.56264 | 0.1771 |
| ADOS Communication | 36 | 2.81 | 1.41 | 18 | 2.67 | 1.14 | 0.69892 |  | - | - | - | - | - | - | - | - |
| ADOS Social Interaction | 36 | 7.81 | 2.75 | 18 | 7.89 | 3.31 | 0.92722 |  | - | - | - | - | - | - | - | - |
| ADOS RRSB | 36 | 2 | 1.22 | 18 | 2.22 | 1.4 | 0.56968 |  | - | - | - | - | - | - | - | - |
| ADOS Total | 36 | 12.61 | 3.65 | 18 | 12.78 | 4.04 | 0.88365 |  | - | - | - | - | - | - | - | - |
| Conners 3 Hyperactive | 32 | 79.84 | 11.5 | 15 | 78.2 | 13.25 | 0.68314 |  | 25 | 47.96 | 10.8 | 16 | 48.31 | 7.43 | 0.90217 | 0 |
| Conners 3 Inattentive | 32 | 76.62 | 11.12 | 15 | 79.2 | 11.6 | 0.47851 |  | 25 | 48.8 | 9.61 | 16 | 49.5 | 10.02 | 0.82599 | 0 |
| Conners Hyperactive | 6 | 65.67 | 14.47 | 4 | 76 | 16.49 | 0.34811 |  | 12 | 46.17 | 4.49 | 4 | 45.75 | 3.5 | 0.85397 | 0.00078 |
| Conners Inattentive | 6 | 69.5 | 7.94 | 4 | 76 | 16.67 | 0.50843 |  | 12 | 45.08 | 4.5 | 4 | 44.5 | 1.73 | 0.71463 | 3.00E-05 |
| Conners + Conners 3 Hyper. | 36 | 78.33 | 12.85 | 18 | 78.83 | 13.01 | 0.89443 |  | 12 | 44.67 | 4.66 | 4 | 43.5 | 2.38 | 0.52952 | 0 |
| Conners + Conners 3 Inatt. | 36 | 76.33 | 10.68 | 18 | 78.28 | 11.29 | 0.54792 |  | 34 | 47.24 | 9.4 | 20 | 47.8 | 6.83 | 0.8003 | 0 |

Participants had either WISC-IV or WISC-V assessments. FSIQ is a score that collapses across the measures. This was required due to the “matchit” function not allowing variables with missing data. This reason is also why we have Conners + Conners 3 Hyper and Conners + Conners 3 Inat (which are just scores collapsed across the Conners and Conners 3^rd^ edition). WISC = Weschler Intelligence Scale for Children, ADOS = Autism Diagnostic Observation Scale, N = number of participants, ASC = Autism Spectrum Condition, TDC = Typically Developing Controls.

As described in the main text, we were aware that the primary statistical approach (i.e., 2-way and 3-way ANOVAs) could result in Type II errors. For this reason, we also compared autistic males and females directly. Supplementary Table 3 on the next page contains relevant statistics from group comparisons made using Welch’s t-tests. Change in performance between protocol pairs was also comparable between autism and controls.

**Supplementary Table 3.**

| Dependent variable | Mean difference | Mean ASC-F | Mean ASC-M | t | df | Lower CI | Upper CI | p |
| --- | --- | --- | --- | --- | --- | --- | --- | --- |
| Mean Simple Response Time (SRT) | 67.197 | 446.468 | 379.271 | 0.862 | 8.771 | -109.921 | 244.315 | 0.412 |
| Mean Choice Response Time (CRT) | 62.897 | 887.61 | 824.713 | 0.379 | 7.91 | -320.987 | 446.781 | 0.715 |
| Static Detection Threshold (SDT) | 0.34 | 7.04 | 6.7 | 0.231 | 9.767 | -2.955 | 3.635 | 0.822 |
| Dynamic Detection Threshold (DDT) | -0.313 | 8.558 | 8.871 | -0.29 | 9.283 | -2.743 | 2.116 | 0.778 |
| Amplitude discrimination threshold (ADT) | 44.125 | 87 | 42.875 | 2.174 | 5.197 | -7.454 | 95.704 | 0.08 |
| ADT with single-site adaptation | 53.5 | 126 | 72.5 | 1.905 | 8.344 | -10.795 | 117.795 | 0.092 |
| Simultaneous frequency discrimination threshold (SMFD) | 2.6 | 41.2 | 38.6 | 1.337 | 10.929 | -1.682 | 6.882 | 0.208 |
| Sequential frequency discrimination threshold (SQFD) | 1.497 | 40.24 | 38.743 | 0.548 | 7.983 | -4.807 | 7.801 | 0.599 |
| Temporal order judgement (TOJ) threshold | -22.57 | 81.44 | 104.01 | -0.484 | 4.938 | -142.996 | 97.856 | 0.649 |
| TOJ threshold with carrier (TOJwc) | 72.292 | 123.438 | 51.145 | 1.976 | 2.195 | -72.426 | 217.011 | 0.175 |
| SRT-CRT | -30.561 | 106.751 | 137.312 | -0.73 | 7.951 | -127.208 | 66.085 | 0.486 |
| SDT-DDT | -32.636 | 25.905 | 58.541 | -1.058 | 8.094 | -103.635 | 38.364 | 0.321 |
| ADT-ADTssa | -86.962 | 57.465 | 144.427 | -0.908 | 9.98 | -300.485 | 126.56 | 0.385 |
| SMFD-SQFD | 1.282 | 3.66 | 2.379 | 0.158 | 8.23 | -17.336 | 19.899 | 0.878 |
| TOJ-TOJwc | 78.225 | 109.512 | 31.287 | 0.528 | 2.977 | -395.513 | 551.963 | 0.634 |

Legend: ASC = Autism Spectrum Condition, ASC-F = autistic females, ASC-M = autistic males, CI = confidence interval. Note that p values are uncorrected for multiple comparisons. Change in performance across condition pairs was estimated for all condition pairs by dividing one condition by the other (e.g., SRT/CRT), multiplying the resultant value by 100 and then subtracting 100 (i.e., representing percentage change).
